# The TNF/TNFR2 signaling pathway is a key regulatory factor in endothelial progenitor cell immunosuppressive effect

**DOI:** 10.1186/s12964-020-00564-3

**Published:** 2020-06-16

**Authors:** Sina Naserian, Mohamed Essameldin Abdelgawad, Mazdak Afshar Bakshloo, Guillaume Ha, Nassim Arouche, José L. Cohen, Benoît L. Salomon, Georges Uzan

**Affiliations:** 1 INSERM UMR-S-MD 1197, Hôpital Paul Brousse, Villejuif, France; 2 CellMedEx, Saint Maur Des Fossés, France; 3 Paris-Saclay University, Villejuif, France; 4 Biochemistry Division, Chemistry department, Faculty of Science, Helwan University, Cairo, Egypt; 5 Univ Paris Est Creteil, INSERM, IMRB, F-94010 Creteil, France; 6 AP-HP, Hopital Henri Mondor, Centre d’investigation clinique biothérapie, F-94010 Creteil, France; 7 Sorbonne Université, INSERM, CNRS, Centre d’Immunologie et des Maladies Infectieuses (CIMI-Paris), Paris, France

**Keywords:** Endothelial Progenitor Cells (EPCs), Endothelial Colony Forming Cells (ECFCs), T cells, Immune regulation, Immune-checkpoint, TNFα/TNFR2 signaling pathway, Cancer immune therapy

## Abstract

**Background:**

Endothelial progenitor cells (EPCs) are non-differentiated endothelial cells (ECs) present in blood circulation that are involved in neo-vascularization and correction of damaged endothelial sites. Since EPCs from patients with vascular disorders are impaired and inefficient, allogenic sources from adult or cord blood are considered as good alternatives. However, due to the reaction of immune system against allogenic cells which usually lead to their elimination, we focused on the exact role of EPCs on immune cells, particularly, T cells which are the most important cells applied in immune rejection. TNFα is one of the main activators of EPCs that recognizes two distinct receptors. TNFR1 is expressed ubiquitously and its interaction with TNFα leads to differentiation and apoptosis, whereas, TNFR2 is expressed predominantly on ECs, immune cells and neural cells and is involved in cell survival and proliferation. Interestingly, it has been shown that different immunosuppressive cells express TNFR2 and this is directly related to their immunosuppressive efficiency. However, little is known about immunological profile and function of TNFR2 in EPCs.

**Methods:**

Using different in-vitro combinations, we performed co-cultures of ECs and T cells to investigate the immunological effect of EPCs on T cells. We interrupted in the TNFα/TNFR2 axis either by blocking the receptor using TNFR2 antagonist or blocking the ligand using T cells derived from TNFα KO mice.

**Results:**

We demonstrated that EPCs are able to suppress T cell proliferation and modulate them towards less pro-inflammatory and active phenotypes. Moreover, we showed that TNFα/TNFR2 immune-checkpoint pathway is critical in EPC immunomodulatory effect.

**Conclusions:**

Our results reveal for the first time a mechanism that EPCs use to suppress immune cells, therefore, enabling them to form new immunosuppressive vessels. Furthermore, we have shown the importance of TNFα/TNFR2 axis in EPCs as an immune checkpoint pathway. We believe that targeting TNFR2 is especially crucial in cancer immune therapy since it controls two crucial aspects of tumor microenvironment: 1) Immunosuppression and 2) Angiogenesis.

Video Abstract. (MP4 46355 kb)

## Background

Circulating Endothelial Progenitor Cells (EPCs) are non-differentiated endothelial cells (ECs) first isolated from adult blood [1]. They are able to integrate vascular structures at damaged or neo-vascularization sites where they differentiate into mature ECs [2, 3], and are then crucial for maintaining the vascular integrity [4, 5]. According to first colony appearance time, two distinct cell populations of EPCs have been identified: early EPCs or Colony Forming Unit-Endothelial Cells (CFU-ECs) and late EPCs or Endothelial Colony Forming Cells (ECFCs) [6]. In-vivo, ECFCs can form stable vessels through incorporating into vascular networks [7–9]. Therefore, ECFCs are accepted as true EPCs progeny expressing EC markers, displaying features of stem/progenitor cells and having high clonogenicity and proliferation rate [10].

ECFCs can be isolated from umbilical cord blood (CB-ECFCs) [11]. We have demonstrated that these ECFCs give rise to higher number of colonies and can be extensively expanded in-vitro compared to ECFCs derived from adult peripheral blood (APB-ECFCs) and their initial clonogenic potential is predictive of their further properties [12]. Furthermore, unlike adult vascular ECs, ECFCs have not yet acquired specialized functions. In presence of appropriate external stimuli, CB-ECFCs acquire properties of specialized ECs such as brain microvascular or arterial ECs [13]. Therefore, ECFCs still bear stem cell features and this could potentially influence their immunogenic properties.

Inflammatory signaling pathways are crucial for the migration of ECs and initiating angiogenesis [14]. It is known that tumor necrosis factor alpha (TNFα) is a pro-inflammatory mediator that could modulate both pro- and anti-angiogenic properties [15–17]. This dual effect is directly related to the concentration of TNFα and duration of exposure [18]. TNFα interacts with two distinct transmembrane receptors, TNFR1 and TNFR2. TNFR1 is expressed on almost all cell types and its binding with TNFα leads to cell death and apoptosis. TNFR2 is strictly expressed on limited cells such as immune cells, ECs, neural cells and Mesenchymal Stem Cells (MSCs) and its interaction with TNFα leads to cell survival and proliferation [19–21]. Through binding to its receptors, TNFα induces and enhances the expression of many pro-angiogenic factors like vascular endothelial growth factor (VEGF), basic fibroblast growth factor (bFGF), and interleukin-8 (IL-8) in ECs [22–24]. Indeed, TNFα/TNFR2 axis supports pro-angiogenic and protective mechanisms and inversely TNFα/TNFR1 axis is involved in deleterious mechanisms. For example, unlike TNFR1 that mediates myocardiac ischemic injuries and exert a toxic effect in myocardial infraction models [25, 26], TNFα/TNFR2 signaling pathway is protective in adult infract myocardium [27], heart ischemic injuries [28] and aging [29, 30]. It has been demonstrated in-vivo that ECFC cell survival, mobilization, differentiation, VEGF expression and ultimately ischemia-induced collateral vessel development depend on TNFα/TNFR2 signaling pathway [30]. Furthermore, unlike TNFR2 KO mice, endothelial cell specific transgenesis of TNFR2 revealed a significant promotion in arteriogenesis and angiogenesis in mice [31].

These findings highlight the important implications of ECFCs in conditions which angiogenesis and neo-vascularization are involved, like in cardiovascular disorders, transplantation and specially cancer. However, little is known about their immunogenicity and the interaction of immune system with these cells in the context of cancer or while administered in an allogenic setting. We have recently demonstrated in a model of bio-artificial vessel that human CB-ECFCs implanted in a microfluidic chambers were able to suppress allogenic T cells in a dose dependent manner [32]. In addition, we showed that human ECFCs are not only capable of inducing new functional vessels in xenogeneic ischemic immunocompetent mice but are tolerated by host immune system and resistant in several tissues after their first administration [33]. Therefore, we aimed to study the interaction between human ECFCs and mice T cells in order to understand through what mechanisms human ECFCs are tolerated in highly inflammatory xenogeneic mice model and are able to properly exert their immunological and angiogenic properties.

To investigate the difference between the sources of ECFCs (cord or adult blood) and the mechanism they use to exert their immunosuppressive effect, we considered the following facts: 1) ECFCs are among the rare cells expressing TNFR2 and TNFα is extremely important for their activation, migration and angiogenic activities [14, 30]. 2) We and others have shown that many immunosuppressive cells including regulatory T cells (T regs), regulatory B cells (B regs) and myeloid derived suppressive cells (MDSCs) express TNFR2 and this is directly related to their immunosuppressive function [34–36]. Therefore, we aimed to investigate 1) the exact effect of ECFCs on T cells, since they are the first line responsible of immunological rejection and 2) if the TNFα/TNFR2 signaling pathways is important for ECFC immunosuppressive function.

We have compared the effect of CB-ECFCs and APB-ECFCs to differentiated adult human aortic endothelial cells (HAEC) on T cells. We evaluated the proliferation capacity, activation profile and cytokine secretion of both CD4 and CD8 T cells after co-culturing with those ECs. Finally, we interfered in the TNFα/TNFR2 axis and demonstrated for the first time that this signaling pathway is critical in ECFC immunomodulatory effect.

## Methods

### ECFC and HAEC isolation and culture

Human samples were used in compliance with the declaration of Helsinki. CB samples from healthy full term newborns were obtained from the CB Bank of St Louis Hospital (Paris, France) which is authorized by the French Regulatory Authority (no. PPC51). Human APB from healthy male adults was obtained from the French Establishment of Blood (EFS, authorization 14/5/011). Legal age to give blood ranges from 18 to 70 years. This activity was declared to and authorized by the French Ministry of Research under number AC- 2008-376, and to the French Organization for standardization under number 201/51848.1. Mononuclear cells (MNC), obtained by density gradient centrifugation, were seeded onto rat-tail collagen type-I (BD-Bioscience) coated wells as previously described [13]. ECFC colonies appeared after 7–20 days of culture. From passage 1 (P1), cells were seeded at 5000 cells/cm2 and grew in EGM-2MV medium + EGM^TM^-2 Endothelial SingleQuots^TM^ Kit (Lonza), referred to hereafter as EGM2 medium.

HAECs were purchased from Lonza and seeded at 5000 cells/cm2 and grew in EGM2 medium for further passages.

### T cell isolation and culture

 Pan T cell isolation kit II (Miltenyi) was used to isolate total CD3^+^ T cells from the spleens of 6 to 12 week old female C57BL/6 mice WT (Envigo and Charles River Laboratories) or TNFαKO mice (B6.129S-Tnf^tm1Gkl^/J, The Jackson Laboratory). Furthermore, CD25^+^ cells were depleted from the CD3^+^ T cell population using anti-CD25 biotin conjugated antibody (7D4, BD-Biosciences), followed by anti-biotin microbeads staining (Miltenyi). Then, the magnetic-activated cell sorting (MACS) method was used in all cell isolations. The resulting CD3^+^CD25^−^ T cells, more than 92% purity, were cultured in the presence of ECs (ECFCs or HAECs). The isolation of T cells from co-culture in presence of ECs is based on the biological capacity of ECs to adhere to plastic plates, however, T cells stay in suspension, hence, we collected them with gentle aspiration.

### In-vitro study design

#### We designed the following experimental conditions:

1. CB-ECFCs + T cells condition: freshly isolated CD3^+^CD25^−^ T cells were added to more than 80% confluent human CB-ECFCs (P3 to P6).
2. APB-ECFCs + T cells condition: freshly isolated CD3^+^CD25^−^ T cells were added to more than 80% confluent human APB-ECFCs (P3 to P5).
3. HAECs + T cells condition: freshly isolated CD3^+^CD25^−^ T cells were added to more than 80% confluent HAECs (P3 to P6).
4. Control condition: freshly isolated CD3^+^CD25^−^ T cells were cultured alone.

### Co-culture of T cells and ECs

ECs (APB-ECFCs, CB-ECFCs and HAECs) were seeded in 6 or 12 well plates and incubated for 3 h in EGM2 medium. Then, mouse CD3^+^CD25^−^ T cells were added to the ECs at different doses depending on experimental conditions in RPMI medium containing 10% FBS, 1% HEPES buffer, and 1% penicillin/streptomycin/neomycin, referred to hereafter as RPMI medium. All co-culture experiments were performed in 50% EGM2 and 50% RPMI. (Hydrocortisone was removed from singlequote of EGM2 medium due to its immunosuppressive effect). In order to stimulate or block the TNFR2 signaling pathway, we used 1 ng/ml of recombinant-human-TNFα (R&D system) or 2 μg/mL human-TNFR2/CD120b/TNFRSF1B neutralizing antibody (Sino Biological). This Antibody does not have cross-reactivity with mouse TNFR2. T cells were collected after 1 or 3 days of co-culture depending on the experimental condition.

### Proliferation assay

This test was conducted in 12-well plates (Falcon). 5 × 10^4^ ECs (APB- ECFCs, CB- ECFCs or HAECs) were co-cultured with 6 increasing doses of mouse CD3^+^CD25^−^ responder T cells in a total volume of 2 ml. The doses were 1/1, 1/2, 1/4, 1/8, 1/16, 1/32 (ECs/T cells). 10^5^ CD3^+^CD25^−^ T cells were used as control T cells alone. T cells were stained with Carboxyfluorescein succinimidyl ester (CFSE) (ThermoFisher) and stimulated by Dynabeads mouse T-activator CD3/CD28 (Gibco) according to provided protocol. After 3 days, T cells were collected and immunostained and the percentage of CFSE^+^ cells was measured among CD4^+^ and CD8^+^T cells.

### T cell activation and cytokine measurement

5 × 10^4^ ECs (APB- ECFCs, CB- ECFCs or HAECs) were co-cultured with 3 × 10^5^ (1/6 ratio) of mouse CD3^+^CD25^−^ T cells in a total volume of 2 ml. CD3^+^CD25^−^ T cells were then stimulated by Dynabeads mouse T-activator CD3/CD28 (Gibco) in compliance with provided protocol. According to proliferation assay, 1/6 is the ratio which CB-ECFCs suppress 50% of responder T cell proliferation. After either 1 or 3 days, CD3^+^CD25^−^ T cells were collected and proceeded with immunostaining. 3 × 10^5^ freshly isolated CD3^+^CD25^−^ T cells were used as control T cells alone. For intracellular cytokine staining, cells were stimulated with 1 μg/mL PMA and 0.5 μg/mL Ionomicyn (Sigma) for 4 h + 1 h with 1 μL/mL GolgiPlug (BD-Biosciences).

### Flow cytometry

Cells were immunostained with the following mAbs: CD31-FITC, CD144-vioblue, KDR-PE-Vio770, CD4-FITC, APC and Vioblue, Foxp3-APC, CD62L-PE, ICOS–PE, CTLA4–biotin or PE, IFNγ–APC, TNFα-FITC or PE, IL-10-APC, IL-17-PE, IL-2-FITC, HLA-G-PE, TGFβ-biotin, REA-control-APC, PE, PE-Cy5 and PE-Cy7, CD8α-FITC or Percp or PE-Cy7 and TNFR2-APC and PE (Miltenyi) streptavidin-PE-Cy7 or PE-Cy5, Foxp3-PE-Cy5, CD25-PE-Cy7 (eBioscience) and TGFβ-PE (Biolegend). Intracellular Foxp3 staining was performed according to the manufacturer’s instructions, using Foxp3 staining buffer set from eBioscience. Events acquired on a LSRFORTESSA flow cytometer (BD-Biosciences) and analyzed using FlowJo software v10 (FlowJo-LLC).

### Statistical analysis

Prism (GraphPad) was used for statistical analysis. Shapiro-Wilk normality test was performed to assess the normal distribution of data. Student *t* test or 1-way ANOVA with post hoc analysis was performed depending on the number of comparatives. For cytometry analysis, we have normalized the MFI values with T-cell alone control group. Then we used unpaired, 2-tailed Student *t* tests or 1-way ANOVA for *P* value generation.

## Results

### ECFCs suppress T cell proliferation

**Fig. 1:**
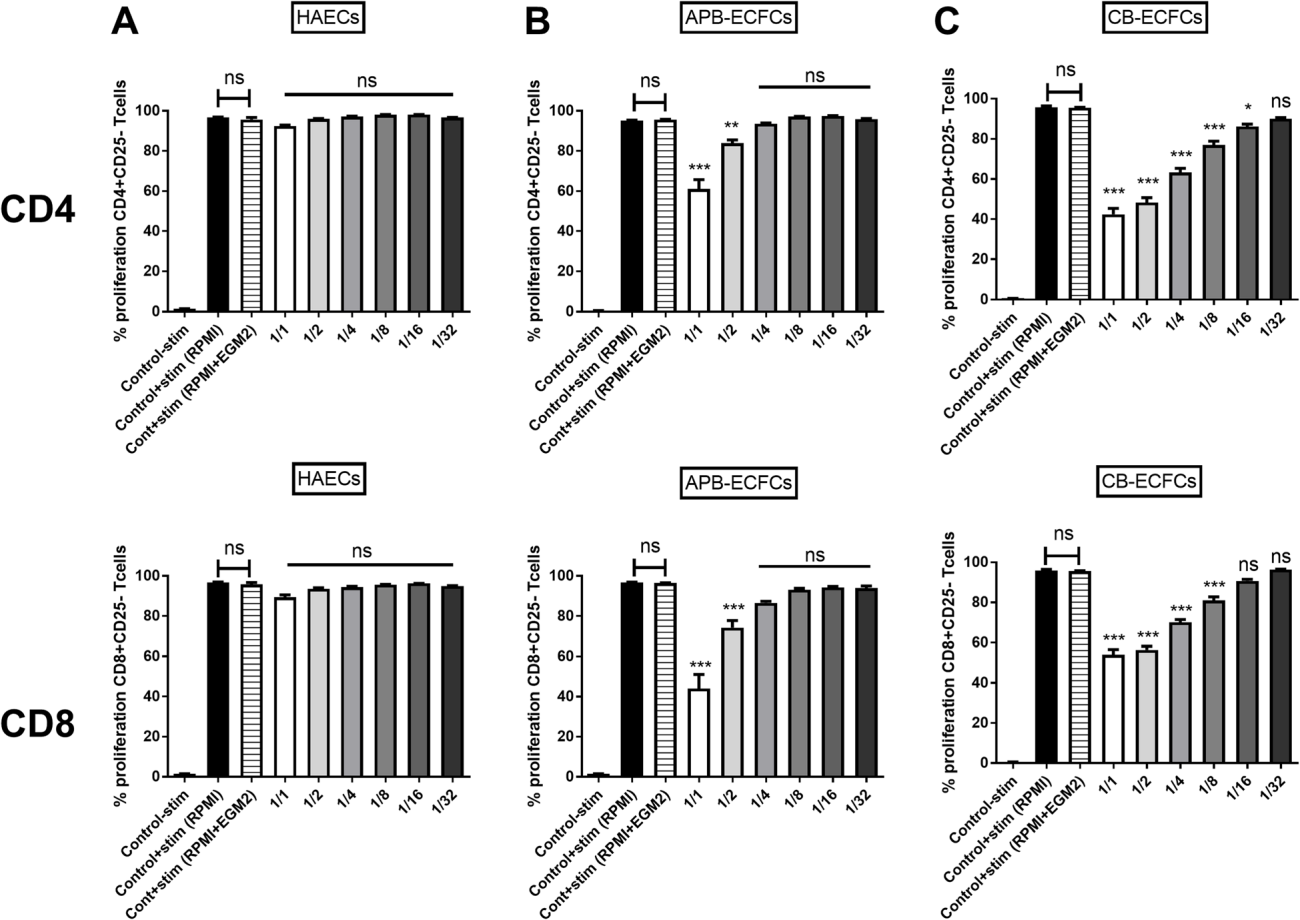
ECFCs can suppress T cell proliferation. Activated CFSE^+^CD3^+^CD25^−^ effector T cells (responder cells) were co-cultured with (**a**) HAECs, (**b**) APB-ECFCs and (**c**) CB-ECFCs in different ECs/T cells ratios (*n* = 8). Proliferation of CD4^+^ T cells (upper graphs) and CD8^+^ T cells (lower graphs) was measured by flow cytometry. The first bar represents the unstimulated T cells alone (*n* = 8), the second bar represents the anti-CD3/CD28 stimulated T cells alone in RPMI medium (*n* = 8), while the third bar is the anti-CD3/CD28 stimulated T cells alone in 50% RPMI+ 50% EGM2 media (*n* = 8). Data are represented as mean value ± SEM collected from 3 independent experiments. One way ANOVA analysis was performed to generate *P* values. ns: non-significant, *P < .05, **P < .01, ***P < .001. Stim: Anti-CD3/CD28 activation Beads

We first investigated the immunogenic effect of undifferentiated ECFCs on T cells compared to differentiated HAECs. CB-ECFCs, ABP-ECFCs and HAECs were co-cultured with CFSE labeled mouse CD3^+^CD25^−^ responder T cells in 6 different ratios (1/1 to 1/32 for ECs/T cells). CD25^+^ T cells were depleted from starting T cell population to eliminate 1) activated T cells and 2) unspecific immunosuppression by T regs. After 3 days of co-culture, total T cells were collected (cells in suspension). The proliferation capacity of two main sub-populations of T cells (CD4^+^ and CD8^+^ T cells) was then studied. Since, two different media are used for T cells (RPMI medium) and ECs (EGM2 medium); we used 50% of each medium in co-culture. To observe the effect of EGM2 medium on T cells, two control group were added in which T cells alone were cultured either in 100% RPMI medium or in 50% EGM2+ 50% RPMI media. No difference was observed between those controls throughout the entire experiments (Fig. 1). Likewise, the co-culture of HAECs with T cells did not change the proliferation capacity of neither CD4^+^ nor CD8^+^ responder T cells regardless of different ratio conditions (Fig. 1a, Sup Figure 1). However, we observed a significant decrease in proliferation capacity of both CD4^+^ and CD8^+^ T cells while co-cultured with APB-ECFCs (Fig. 1b, Sup Figure 1). The significant immunosuppressive effect was only observed in 1/1 and 1/2 ratios (34.12 and 11.2% of suppression, respectively) for CD4^+^ T cells and equally for CD8^+^ T cells (52.65 and 22.55% of suppression, respectively) and then was lost for more elevated doses of T cells (Fig. 1b). An even stronger dose dependent immunosuppression of T cells was found while co-cultured with CB-ECFCs, starting from 1/1 (53.6% of suppression) up to 1/16 (9.69% of suppression) ratio for CD4^+^ T cells and from 1/1 (41.84% of suppression) up to 1/8 ratios (15% of suppression) for CD8^+^ T cells (Fig. 1c, Sup Figure 1). Hence, we report a remarkable dose dependent immunosuppressive effect of ECFCs on T cells which is not observed in other differentiated ECs such as HAECs. Moreover, we demonstrate that this immunosuppressive effect was more accentuated in CB-ECFCs compared to APB-ECFCs.

### ECFCs modulate CD4^+^ T cell activation markers

**Fig. 2:**
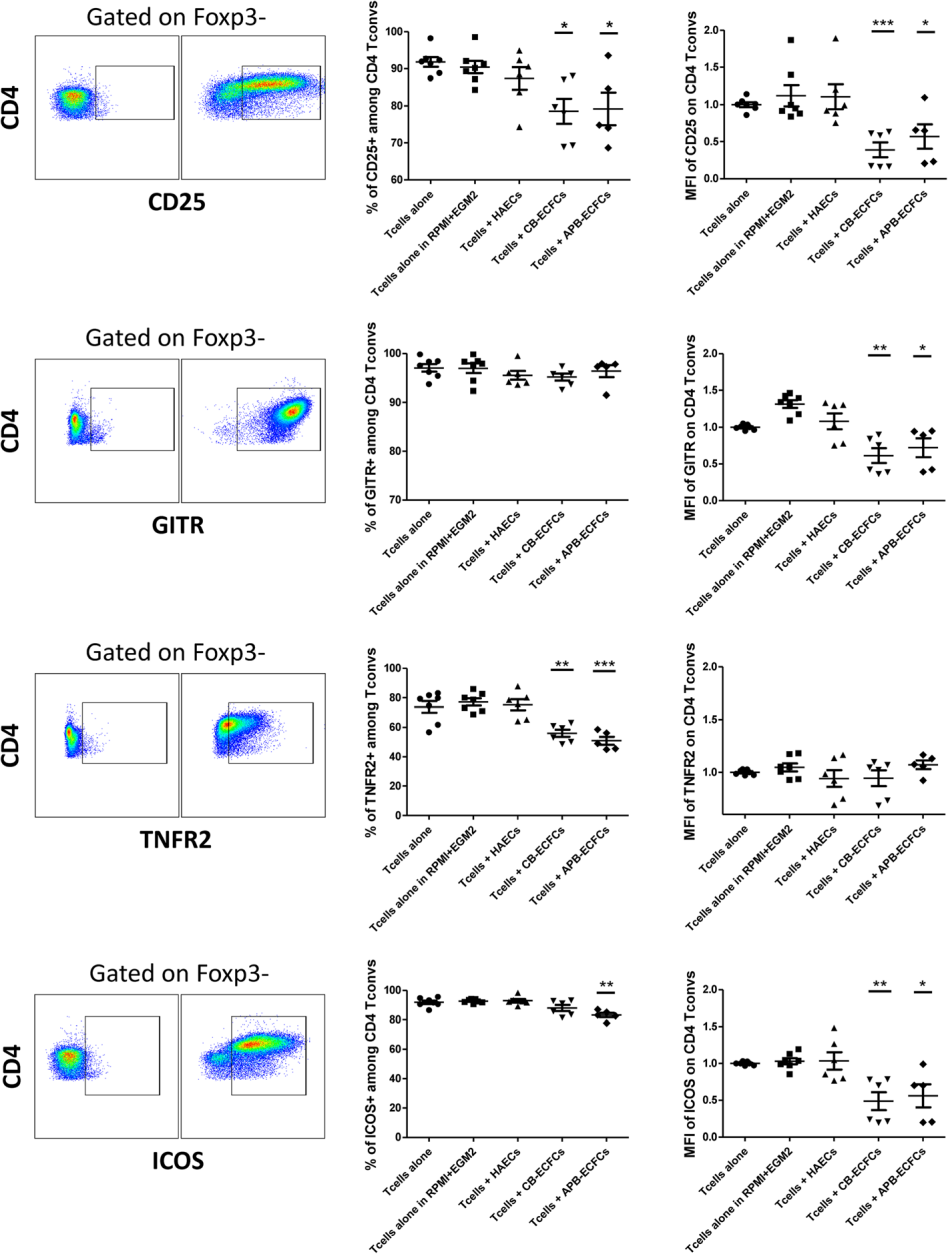
ECFCs can modulate CD4^+^ T cell activation markers. Anti-CD3/CD28 activated CD3^+^CD25^−^ effector T cells were co-cultured with HAECs, CB-ECFCs and APB-ECFCs in fixed 1/6 ratio. After 3 days, T cells were collected and different activation markers were studied. Cells were gated on CD4^+^Foxp3^−^ conventional T cells. For each marker the strategy of gating is indicated on the left and below the figure. The representative gating panels on the left demonstrate fluorescence minus one (FMO) controls. Each dot represents a measured value (*n* = 7 for Tcells alone, Tcells alone in RPMI+EGM2 media and *n* = 6 for Tcells + HAECs, Tcells + CB-ECFCs and *n* = 5 for Tcells + APB-ECFCs) collected from 2 independent experiments. For each group of values, horizontal lines represent mean value and standard error of the mean. MFI values have been normalized with T cells alone control group. One way ANOVA analysis was performed to generate *P* values. ns: non-significant, *P < .05, **P < .01, ***P < .001

We then investigated if ECFCs are able to modulate the activation profile of both CD4^+^ and CD8^+^ T cells. Therefore, HAECs, CB-ECFCs and APB-ECFCs were co-cultured with mouse CD3^+^CD25^−^ T cells for periods of 1 and 3 days since the first few days after transplantation is crucial in case of inflammatory responses [37]. Total T cells were harvested and further analyzed for the percentage of expression and mean fluorescence intensity (MFI) of different activation markers among conventional CD4^+^ Foxp3^−^ T cells (CD4^+^ T convs). We first measured the expression of CD25; α chain of the IL-2 receptor, constitutively expressed on T regs and activated T cells [38, 39]. After 3 days, we observed a dramatic decrease of the percentage of CD25^+^ cells and CD25 expression level among CD4^+^ T convs, only when co-cultured with CB-ECFCs or APB-ECFCs and not with HAECs (Fig. 2). Moreover, we evaluated the expression of two members of TNFα receptor superfamily, GITR (TNFRSF18) and TNFR2 (CD120b or TNFRSF1B). Three days after co-culture, we observed a remarkable decrease in expression level of GITR and percentage of expression of TNFR2 marker among CD4^+^ T convs, only when co-cultured with CB-ECFCs or APB-ECFCs and not with HAECs (Fig. 2). Finally, we studied the expression of inducible co-stimulatory molecule (ICOS) among CD4^+^ T convs. It was shown that ICOS co-stimulatory receptor is essential for T cell activation and proliferation [40]. After 3 days of co-culture, we observed a significant reduction in percentage of ICOS^+^ cells among CD4^+^ T convs only in co-culture condition with APB-ECFCs and a decrease in expression level after co-culture with both CB-ECFCs and APB-ECFCs and never with HAECs (Fig. 2). All the mentioned activation markers were also measured on CD4^+^ T convs, 1 day after co-culture with different ECs. We did not observe a significant alter in the percentage of expression of none of those markers; however, the MFI of all T cell activation markers was dramatically decreased only when co-cultured with ECFCs (Sup Figure 2). No significant difference was noticed between CB-ECFCs and APB-ECFCs in regard to downregulation of measured markers. Altogether, these data suggest that unlike differentiated ECs, ECFCs from both CB and APB sources are able to strongly down-modulate CD4^+^ T cells activation which is in accordance with their less proliferative capacity (Fig. 1).

### ECFCs modulate CD8^+^ T cell activation markers

**Fig. 3:**
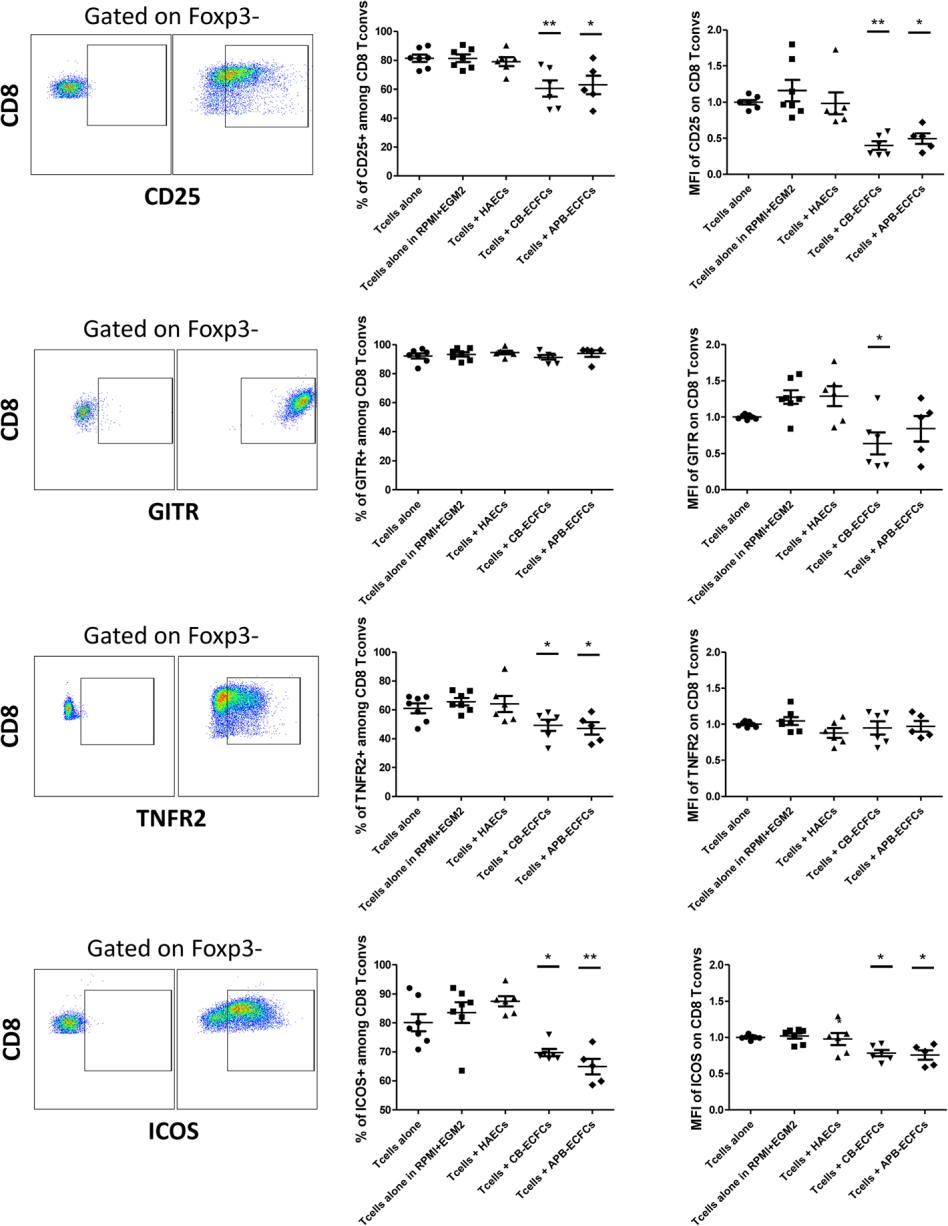
ECFCs can modulate CD8^+^ T cell activation markers. Anti-CD3/CD28 activated CD3^+^CD25^−^ effector T cells were co-cultured with HAECs, CB-ECFCs and APB-ECFCs in a fixed 1/6 ratio. After 3 days, T cells were collected and different activation markers were studied. Cells were gated on CD8^+^Foxp3^−^ conventional T cells. For each marker the strategy of gating is indicated on the left and below the figure. The representative gating panels on the left demonstrate FMO controls. Each dot represents a measured value (*n* = 7 for Tcells alone, Tcells alone in RPMI+EGM2 and *n* = 6 for Tcells + HAECs, Tcells + CB-ECFCs and *n* = 5 for Tcells + APB-ECFCs) collected from 2 independent experiments. For each group of values, horizontal lines represent mean value and standard error of the mean. MFI values have been normalized with T cells alone control group. One way ANOVA analysis was performed to generate *P* values. ns: non-significant, *P < .05, **P < .01, ***P < .001

To investigate the effect of different ECs on cytotoxic T cells, HAECs, CB-ECFCs and APB-ECFCs were co-cultured with CD3^+^CD25^−^ responder T cells for periods of 1 and 3 days. T cells were collected and further analyzed for the percentage of expression and MFI  of different activation markers among conventional CD8^+^ Foxp3^−^ T cells (CD8^+^ T convs). After 3 days, we observed a dramatic decrease of the percentage of CD25^+^ cells and CD25 expression level among CD8^+^ T convs, only when co-cultured with CB-ECFCs or APB-ECFCs and not with HAECs (Fig. 3). This effect was more accentuated in T cells collected from the co-culture of CB-ECFCs as compared to APB-ECFCs. Furthermore, our data revealed a significant decrease in the expression level of GITR only in T cells + CB-ECFCs group and the percentage of expression of TNFR2 among CD8^+^ T convs, only when co-cultured with CB-ECFCs or APB-ECFCs and not with HAECs (Fig. 3). Finally, we noticed a significant reduction in percentage of ICOS^+^ cells and its expression level on CD8^+^ T convs only in co-culture condition with CB-ECFCs and APB-ECFCs and never with HAECs (Fig. 3). All the mentioned activation markers were also measured on CD8^+^ T convs, 1 day after co-culture in presence of different ECs. We did not observe a significant alter in the percentage of expression of CD25 and GITR markers, however, their expression level was remarkably decreased (Sup Figure 3). Moreover, both the percentages of expression and MFI of TNFR2 and ICOS significantly decreased in T cells+CB-ECFCs and T cells+APB-ECFCs group and not in T cells+HAECs group. No significant difference was noticed between CB-ECFCs and APB-ECFCs in regard to downregulation of mentioned activation markers. These data suggest that only CB- and APB-ECFCs are capable of down-modulating CD8^+^ T cells activation which is in accordance with their less proliferative capacity (Fig. 1).

### ECFCs reduce the capacity of T cell pro-inflammatory cytokine production

**Fig. 4:**
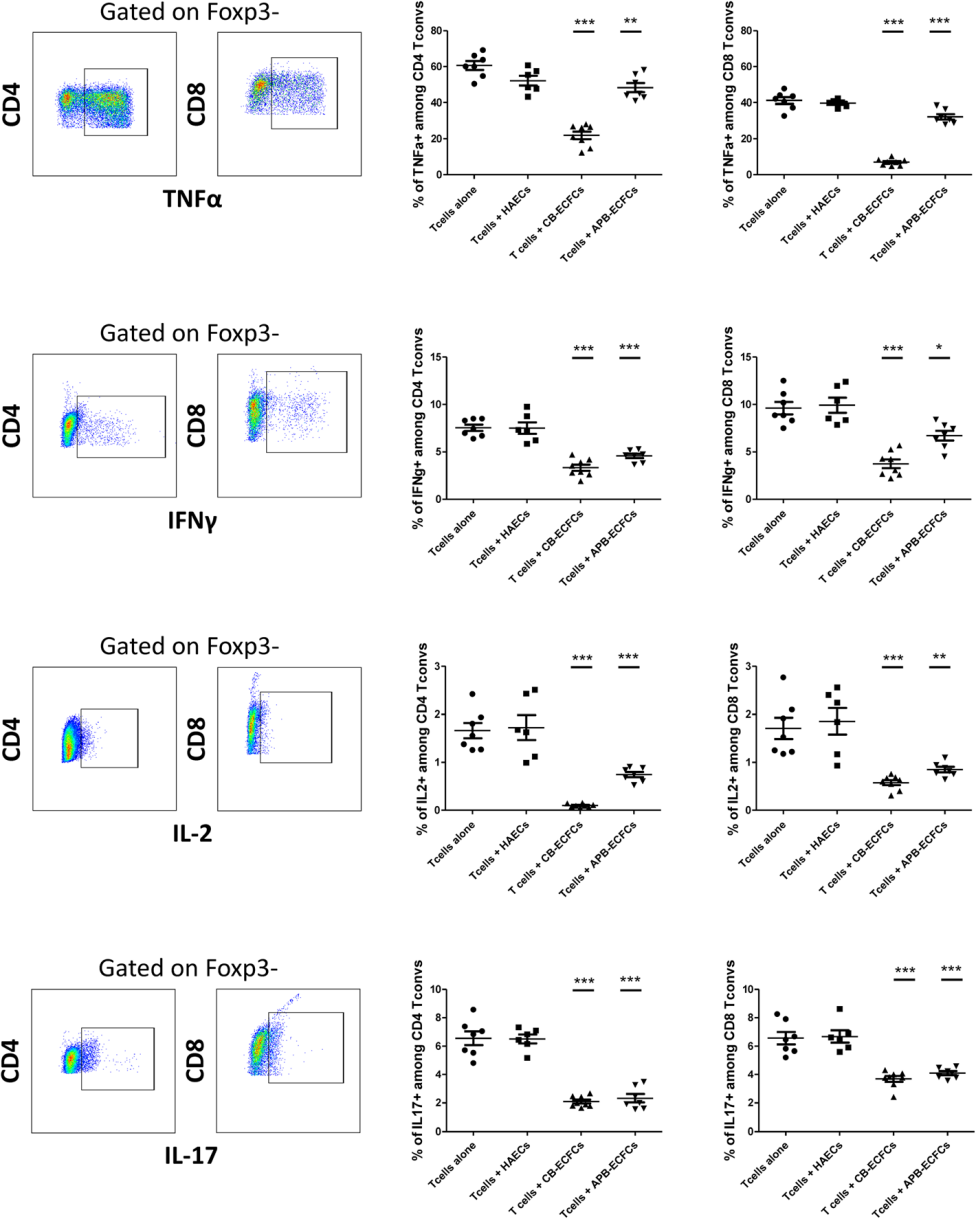
ECFCs can reduce the capacity of T cells to produce pro-inflammatory cytokines. CD3^+^CD25^−^ T cells were co-cultured with HAECs, CB-ECFCs and APB-ECFCs in a fixed 1/6 ratio. After 3 days, T cells were collected, activated with PMA/Ionomycin and then treated with Golgi Plug (a protein transport inhibitor). Intracellular cytokine production was determined in both CD4^+^ (left graphs) and CD8^+^ T cells (right graphs) by flow cytometry. Cells were gated on CD4^+^Foxp3^−^ or CD8^+^Foxp3^−^ T cells. For each marker the gating strategy is indicated on the left and below the figure. Each dot represents a measured value (*n* = 7 for Tcell alone, *n* = 6 for HAEC group, *n* = 8 for Tcells + CB-ECFCs group and *n* = 7 for Tcells + APB-ECFCs group) collected from 2 independent experiments. For each group of values, horizontal lines represent mean value and standard error of the mean. One way ANOVA analysis was performed to generate *P* values. ns: non-significant*P < .05, **P < .01, ***P < .001

After activation, T cells produce anti- or pro-inflammatory cytokines. We examined the effect of ECFCs on T cell cytokine production capacity. We quantified the principle cytokines secreted by four main sub-populations of T helpers (Th1, Th2, Th17 and T reg) and cytotoxic T cells (Tc1, Tc2, Tc17 and T reg). CB-ECFCs, APB-ECFCs and HAECs were co-cultured with mouse CD3^+^CD25^−^ T cells. 3 days after, T cells were collected and analyzed for their cytokine production capacity. We first assessed the ability of T cells to produce anti-inflammatory cytokines. No IL-10 and TGFβ production was observed neither by CD4^+^ nor CD8^+^ T cells (data not shown). Furthermore, we investigated the capacity of T cells to produce pro-inflammatory cytokines. Interestingly, we observed an impressive reduction in production of TNFα, IFNγ, IL-2 and IL-17 both by CD4^+^ and CD8^+^ T convs, merely, after co-culturing with ECFCs and not with HAECs (Fig. 4). Our results did not reveal any difference in cytokine production between T cells alone and T cells+HAECs group. However, the comparison between CB-ECFCs and APB-ECFCs revealed a trend for stronger immunomodulatory effect by CB-ECFCs (Fig. 4).

### ECFC immunosuppressive effect is TNFα/TNFR2 dependent

**Fig. 5:**
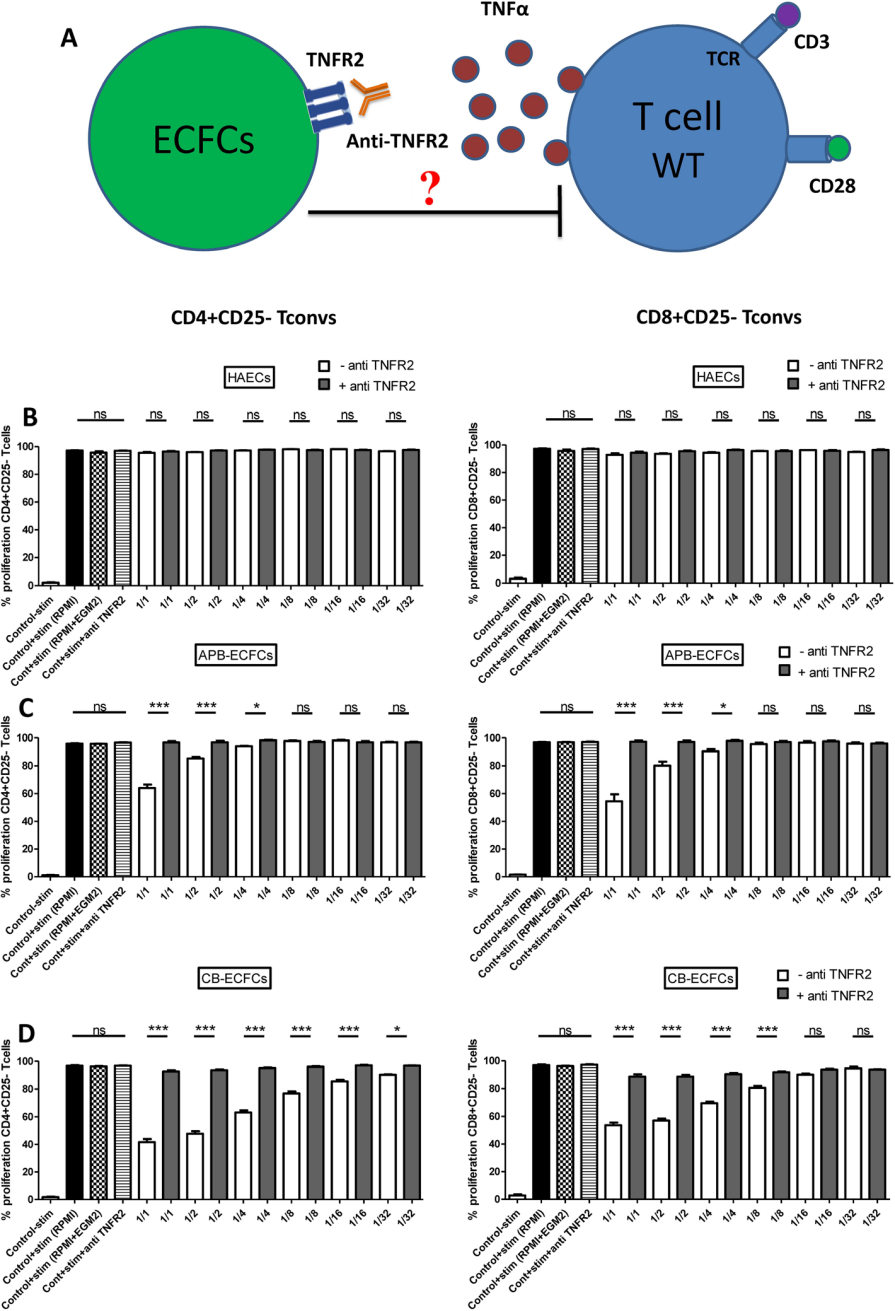
ECFCs immunosuppressive effect is entirely abolished when TNFR2 is blocked on ECFCs. **a** This schematic depicts our hypothesis based on the direct involvement of TNFα/TNFR2 axis in immunomodulatory functions observed by ECFCs. First, we have interfered with this signaling pathway by neutralizing TNFR2 receptor expressed on ECFCs using anti-TNFR2 monoclonal anti-body. In this setting activated WT T cells are producing TNFα but no TNFR2 receptor is available on ECFCs. Anti-CD3/CD28 activated CFSE^+^CD3^+^CD25^−^ effector WT T cells were co-cultured with **b** HAECs, **c** APB-ECFCs and **d** CB-ECFCs in 6 different ECs/T cells ratios (*n* = 8). Proliferation of CD4^+^ CD25^−^ T cells (left graphs) and CD8^+^ CD25^−^ T cells (right graphs) was measured by flow cytometry. The first bar represents the unstimulated T cells alone (Control-stim, *n* = 8), the second bar represents the anti-CD3/CD28 stimulated T cells alone in RPMI medium (Control+stim (RPMI), *n* = 8), the third bar represents the stimulated T cells alone in 50% RPMI+ 50% EGM2 media (Cont+stim (RPMI+EGM2), *n* = 8) and the forth bar represents T cells alone + anti-TNFR2 neutralizing mAb (Cont+anti-TNFR2), *n* = 8). Data are represented as mean value ± SEM collected from 3 independent experiments. One way ANOVA analysis was performed to generate P values. ns: non-significant, *P < .05, **P < .01, ***P < .001. Stim: Anti-CD3 and anti-CD28 activation Beads. TCR = T cell receptor

To understand by which mechanism ECFCs exert their immunosuppressive effect, we investigated if the TNFα/TNFR2 axis is involved in the observed results. We reproduced T cell proliferation assay in co-culture with different ECs while blocking the TNFα/TNFR2 signaling pathway via treatment by anti-TNFR2 neutralizing antibody (anti-TNFR2 mAb). In this setting, TNFα produced by activated T cells (membrane and secreted forms) will not interact with TNFR2 expressed by ECFCs (Fig. 5a). We noticed that blocking TNFR2 had no impact neither on CD4^+^ nor CD8^+^ T cell proliferation when co-cultured with HAECs (Fig. 5b). However, interestingly, the immunosuppressive effect of CB-ECFCs and APB-ECFCs was fully abolished while treated with anti-TNFR2 mAb in all the ratios of both CD4^+^ and CD8^+^ T cells (Fig. 5c and d).

To reinforce our results, we used a second in-vitro model to block TNFα/TNFR2 signaling pathway by using TNFα-deficient T cells harvested from TNFα KO mice. These T cells do not produce any TNFα but ECFCs express TNFR2 (Sup Figure 4a). Again, in the absence of TNFα, neither HAECs nor ECFCs were capable to suppress different T cell populations proving the importance of this signaling pathway (Sup Figure 4b, c, d and e).

### TNFα/TNFR2 signaling pathway regulates ECFC anti-inflammatory cytokine production

**Fig. 6:**
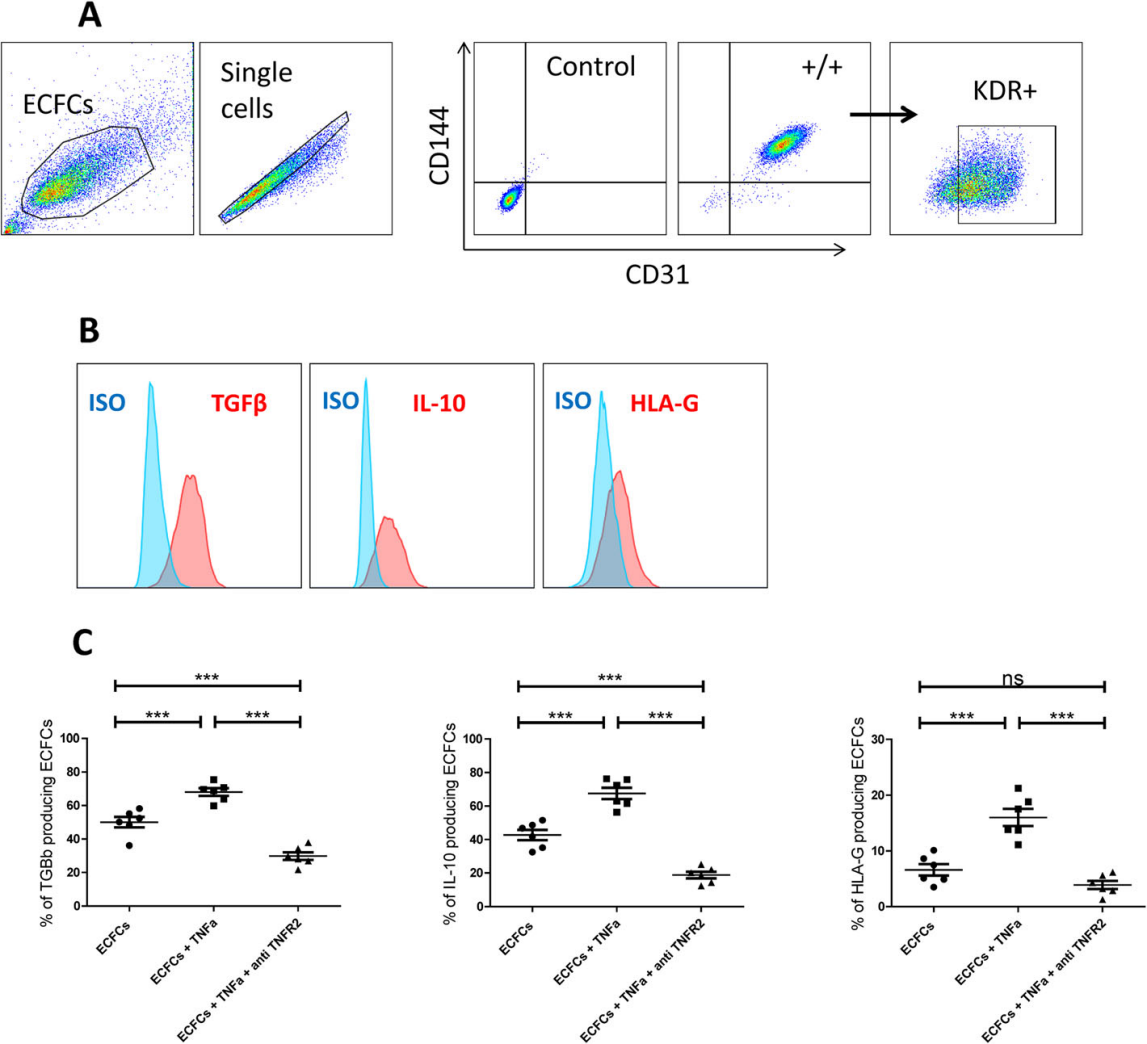
TNFα/TNFR2 signaling pathway can regulate anti-inflammatory cytokine production by ECFCs. **a** A representative of the gating strategy for selecting CD31^+^CD144^+^KDR^+^ ECFCs. **b** A representative of flow cytometry analysis depicting ECFC’s capacity to produce anti-inflammatory cytokines TGFβ, IL-10 and HLA-G. **c** CB-ECFCs were treated either with TNFα or with anti-TNFR2 neutralizing mAb 1 day prior TNFα addition and their capacity to produce TGFβ, IL-10 and HLA-G production were compared with untreated ECFCs (basal level). Each dot represents a measured value (*n* = 6) collected from 2 independent experiments. For each group of values, horizontal lines represent mean value and standard error of the mean. One way ANOVA analysis was performed to generate *P* values. ns: non-significant, *P < .05, **P < .01, ***P < .001. KDR = CD309 or VEGF receptor type 2, ISO = Isotype control

We have shown that ECFCs are able to produce TGFβ, IL-10 and HLA-G cytokines. TNFα/TNFR2 signaling pathways is an immune-checkpoint that changes the balance of immune response in both directions [19, 35]. It was reported that there is a direct relation between the expression of TNFR2 and increased secretion of IL-10 and TGFβ [36, 41]. Therefore, we investigated if stimulation or blocking TNFR2 signaling pathway could change anti-inflammatory cytokine production by ECFCs. We focused on CD31^+^CD144^+^ KDR^+^ (VEGFR2^+^) CB-ECFCs (Fig. 6a) and confirmed that they indeed produce the anti-inflammatory cytokines TGFβ, IL-10 and HLA-G at basal level (Fig. 6b). Furthermore, we stimulated TNFR2 by addition of 1 ng/ml TNFα or inversely, blocked it using 2μg/ml anti-TNFR2 mAb before adding TNFα. We observed that the addition of TNFα can significantly boost TGFβ, IL-10 and HLA-G production compared to basal level (Fig. 6c). Conversely, using anti-TNFR2 mAb has significantly decreased TGFβ and IL-10 production and lowered HLA-G production (Fig. 6c).

## Discussion

Here, we first compared the immunogenicity of CB-ECFCs and APB-ECFCs to differentiated HAECs using different co-culture conditions with mouse T cells. Unlike HAECs, both CB-ECFCs and APB-ECFCs were immunosuppressive against CD4^+^Foxp3^−^ and CD8^+^Foxp3^−^ T cells in a dose dependent manner. This finding is important since no specific marker is yet discovered to distinguish between EPCs and other ECs, therefor, could be a functional test to characterize them.

We then investigated the effect of ECFCs on activation profile of T cells by quantifying different markers like CD25, ICOS, GITR and TNFR2 on mouse T cells after co-culturing them with human ECs. Again, unlike HAECs, CB-ECFCs and APB-ECFCs decreased all activation markers on both CD4^+^ and CD8^+^ populations, beginning from day 1 and stonger at day 3. Among different T cell activation markers, we targeted two TNFα receptor superfamily members, GITR and TNFR2, expressed by activated T cells [42, 43] and demonstrated a significant decrease in their expression merely by ECFCs and not by HAECs. This reflects a complex modulation of TNFα signaling in T cells in the presence of ECFCs. Our results extend the idea that when TNFR2 is decreased on T cells, they will be more efficiently suppressed by immunosuppressive cells like T regs [43] and here by ECFCs. Finally, to investigate the effect of ECFCs on T cell function, we measured their capacity to produce different cytokines like TGFβ, IL-10, IL-2, IL-17, TNFα and IFNγ. We demonstrated a significant reduction in the percentage of pro-inflammatory cytokine producing T cells co-cultured with CB-ECFCs and APB-ECFCs and not by HAECs. Our first hypothesis was that ECFCs might convert T conv to T regs that are further producing anti-inflammatory cytokines, the mechanism that is widely accepted for MSCs [44–46]. However, we did not find any IL-10 and TGFβ production neither by CD4^+^ nor by CD8^+^ T cells. This is in accordance with the absence of Foxp3 induction in T convs after co-culturing with ECFCs or HAECs (data not shown). Thus, ECFC immunosuppressive effect is not T reg dependent.

These findings suggest that unlike HAECs with destined specialized functions, ECFCs are not yet specialized and there could be a direct relation between stem cell features and the immunoregulatory properties of ECFCs. Accordingly, other studies reported that stem cells derived from neonatal sources are less susceptible to immune rejection compared to adult cells [47].

These results are extremely important in the field of tissue engineering and for creating bio-artificial organs such as bio-artificial vessel and bio-artificial lung which implanted allogenic ECs are in direct contact with patient’s blood. ECFCs from both CB and APB sources are demonstrating immunosuppressive and anti-inflammatory properties against total peripheral blood mononuclear cells [33] and particularly T cells, as proved here; therefore, they could be the ideal EC choice since they could tolerate allogenic responses and avoid immune rejection.

In the next step, we investigated through which mechanism ECFCs exert their immunoregulatory function. It has been shown that effector T cell activation can boost T reg expansion and function, a phenomenon partly dependent on TNFα [48, 49]. In the context of GVHD, we showed that preventing TNF/TNFR2 interaction abolished T reg immunosuppressive effect [35, 50]. In addition, other studies reported that immunosuppressive cells including B reg and MDSCs, also express TNFR2 and this is directly related to their immunosuppressive function [34, 36]. ECs also express TNFR2 and interestingly this expression is higher on ECFCs than on HAECs. Inversely, HAECs express higher TNFR1 levels (Sup Figure 5). Moreover, TNFα is crucial for ECFC physiological function [14, 30, 51]. Thus, we investigated the role of TNFα/TNFR2 signaling pathways on ECFC immunosuppressive function. We interfered in the TNFα/TNFR2 axis and demonstrated for the first time that it is critical in ECFC immunomodulatory effect. Our findings rely on two experimental approaches (blocking with anti-TNFR2 mAb and using TNFα-deficient T cells) and performed on two sources of EPCs (CB-ECFCs and APB-ECFCs).

The expression of TNFR2 on immunosuppressive cells is related to their increased capacity of IL-10 and TGFβ secretion [36, 41]. CB-CD34^+^ cells express different isoforms of HLA-G molecules [52]. Accordingly, we observed that ECFCs produce IL-10, TGFβ and HLA-G anti-inflammatory cytokines even at basal level without any activation. Interestingly, adding TNFα significantly boosted the production of those cytokines. Indeed this effect cannot be TNFR1 dependent since blocking TNFR2 (while only TNFR1 is available) has decreased the production of those cytokines. Inflammatory factors, like IL-1, TNFα and IFNγ, promote MSC and T reg immunoregulatory functions [53–56]. Accordingly, we report that TNFα enhances the ECFC immunomodulation in an inflamed environment. These data demonstrates that TNFR2 is an immune checkpoint molecule playing as a tuning system for ECFCs, enabling regulation of their immunological features. Once TNFR2 is stimulated by its agonist, it boosts the anti-inflammatory profile of ECFCs and once it is blocked by proper antagonist, it hampers that function.

It will be interesting to investigate the in-vivo effect of anti-TNFR2 therapy on formation of new vessels and immunosuppression by ECFCs.

**Fig. 7:**
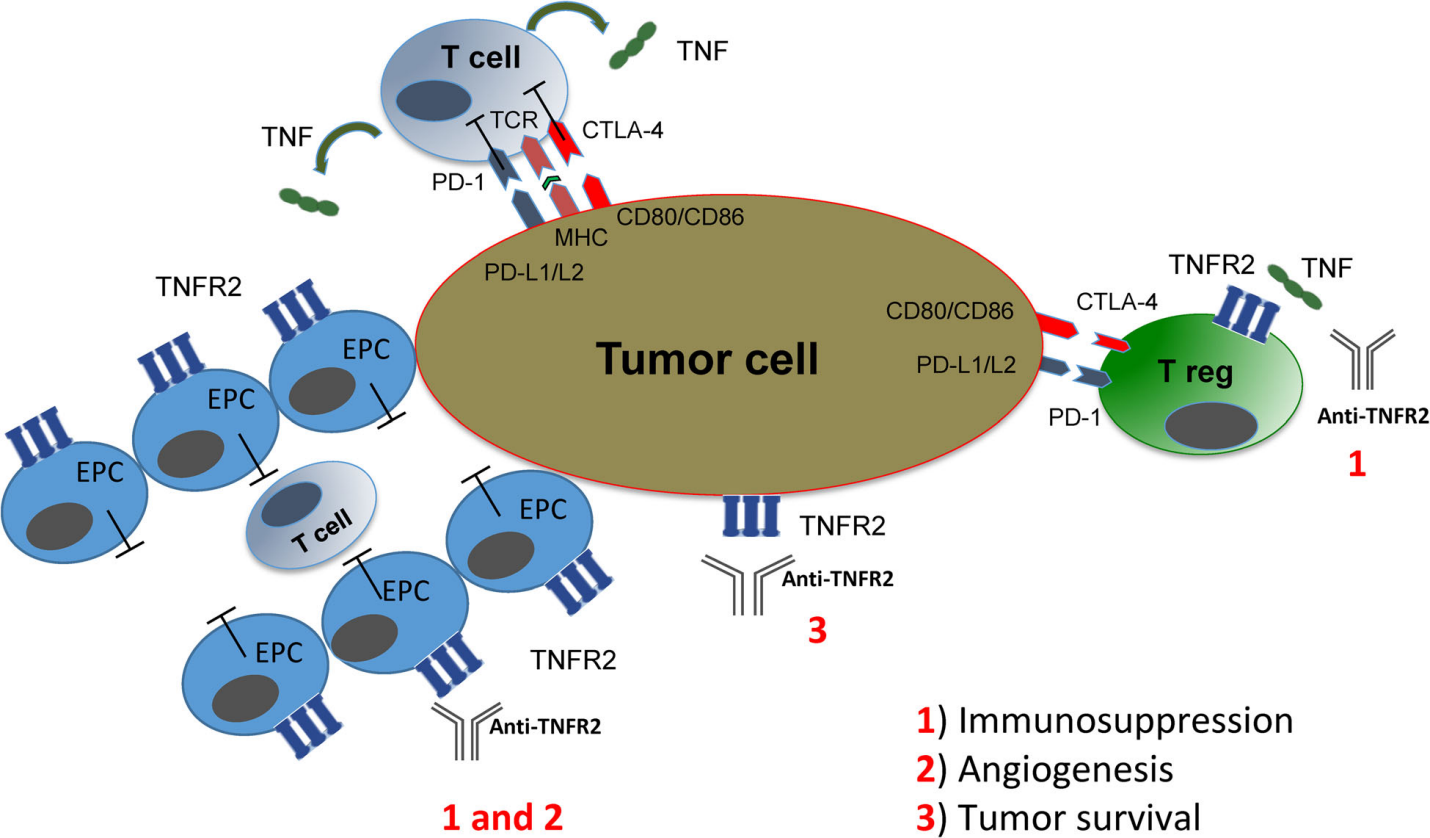
TNFα/TNFR2 immune checkpoint signaling pathway could be used to target different aspects of tumor cells micro environment. This schematic depicts three different possibilities which tumor cells use to recruit/interact with other cells to escape from host immune response and to create highly angiogenic and immunosuppressive microenvironment. Targeting TNFα/TNFR2 axis could first, inhibit Tregs and the consequent immunosuppression. Second, it interrupts tumor survival and proliferation. Third, it hampers angiogenesis and immunosuppression by EPCs. TCR = T Cell Receptor, MHC = Major Histocompatibility Complex, PD1 = Programmed cell death protein 1, PDL1/L2 = Programmed Death-ligand 1, and 2, CTLA-4 = Cytotoxic T-Lymphocyte-Associated protein 4

In cancer, it has been shown that tumor cells through different mechanisms including VEGF and TNFα secretion are able to recruit ECFCs to form new vessels (Fig. 7) [57–60]. It would be interesting to apply anti-TNFR2 treatment and measure tumor neo-vascularization and progression in real time after injection of labelled ECFCs. We are convinced that, blocking the TNF/TNFR2 signaling pathway is truly one stone three birds in cancer microenvironment. First, it inhibits T regs and the consequent immunosuppression. Second, it interrupts tumor survival and proliferation. Recent publications revealed that some tumor cells are also expressing TNFR2 marker and administration of anti-TNFR2 directly eradicated them [61, 62]. Third, and more interesting, it could hamper tumor angiogenesis and immunosuppression caused by EPCs (Fig. 7).

## Conclusions

Here we report that unlike differentiated ECs, their progenitors from both APB and CB sources are demonstrating immunoregulatory properties. This function was more accentuated for CB-EPCs compared to APB-EPCs making them a perfect choice for cell therapy of cardiovascular disorders, tissue engineering, bio-artificial organs and organ on chips manufacturing. Our results reveal for the first time a mechanism that EPCs use to suppress immune cells. Through different in-vitro experimental approaches, we have blocked TNFα/TNFR2 signaling pathway and showed the importance of this immune checkpoint axis in EPC immunoregulatroy function such as production of different anti-inflammatory cytokines like IL-10, TGFβ and HLA-G. Altogether, we believe that targeting TNFR2 using its proper antagonist is an effective way for cancer treatment, since not only it efficiently controls immunosuppression by EPCs and other TNFR2^+^ immunosuppressive cells but also tumor angiogenesis and survival. Inversely, administration of TNFR2 agonist could boost EPC immunoregulatroy function in cases like transplantation that increased immunosuppression and angiogenesis are especially crucial.

## Supplementary information

**Additional file 1: Supplementary Figure 1.** Flow cytometry representative of proliferation assay. **Supplementary Figure 2.** ECFCs can modulate CD4^+^ T cell activation markers. **Supplementary Figure 3.** ECFCs can modulate CD8^+^ T cell activation markers. **Supplementary Figure 4.** ECFCs immunosuppressive effect is entirely abolished when T cells are incapable of TNFα production. **Supplementary Figure 5.** Expression of TNFR1 and TNFR2 on different endothelial cells.

## Data Availability

The datasets used and/or analysed during the current study are available from the corresponding author on reasonable request.

## Abbreviations

APB: Adult Peripheral Blood
B reg: Regulatory B cell
bFGF: Basic Fibroblast Growth Factor
CB: Cord Blood
CFSE: Carboxyfluorescein succinimidyl ester
(CFU-ECs): Colony Forming Unit-Endothelial Cells
ECFCs: Endothelial Colony Forming Cells
ECs: Endothelial Cells
EPCs: Endothelial Progenitor Cells
HAECs: Human Aortic Endothelial Cells
ICOS: Inducible Co-Stimulatory molecule
mAB: Monoclonal Anti Body
MACS: Magnetic-Activated Cell Sorting
MDCSs: Myeloid Derived Suppressive Cells
MFI: Mean Fluorescence Intensity
MNC: Mononuclear cells
MSCs: Mesenchymal Stem Cells
P: Passage
T conv: Conventional T cell
T eff: Effector T cell
T reg: Regulatory T cell
Tc: Cytotoxic T cells
Th: T helper Cells
TNFR1: Tumor Necrosis Factor Receptor 1
TNFR2: Tumor Necrosis Factor Receptor 2
TNFα: Tumor Necrosis Factor alpha
VEGF: Vascular Endothelial Growth Factor
VEGFR2: Vascular Endothelial Growth Factor Receptor 2
