# Supplementary material for: The TNF/TNFR2 signaling pathway is a key regulatory factor in endothelial progenitor cell immunosuppressive effect
Source: Cell Commun Signal. 2020 Jun 16;18:94. doi: 10.1186/s12964-020-00564-3 (PMC7298859; doi:10.1186/s12964-020-00564-3)
Supplement: Supplementary file 2 — Additional file 1: Supplementary Figure 1. Flow cytometry representative of proliferation assay. Supplementary Figure 2. ECFCs can modulate CD4+ T cell activation markers. Supplementary Figure 3. ECFCs can modulate CD8+ T cell activation markers. Supplementary Figure 4. ECFCs immunosuppressive effect is entirely abolished when T cells are incapable of TNFα production. Supplementary Figure 5. Expression of TNFR1 and TNFR2 on different endothelial cells. [file 12964_2020_564_MOESM1_ESM.zip › Supplimental Materials.docx]

**
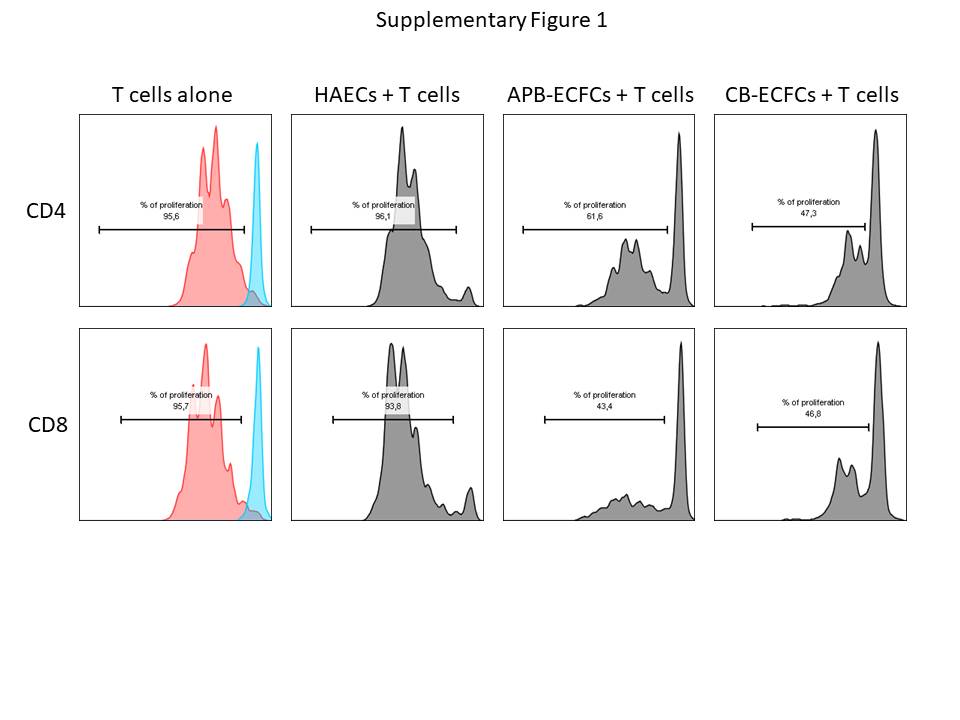
**

**Supplementary Figure 1:**

**Flow cytometry representative of proliferation assay.** Non-stimulated WT CFSE^+^ T cells have a single peak represented in blue. Upon stimulation with anti-CD3/CD28 microbeads, CFSE^+^ T cells alone or in co-culture with different ECs (HAECs, APB-ECFCs and CB-ECFCs) will proliferate. One can see less intensity of CFSE fluorescence in APB-ECFCs + T cells and CB-ECFCs + T cells groups in comparison to HAECs + T cells group, interpreted as less proliferation capacity.

**
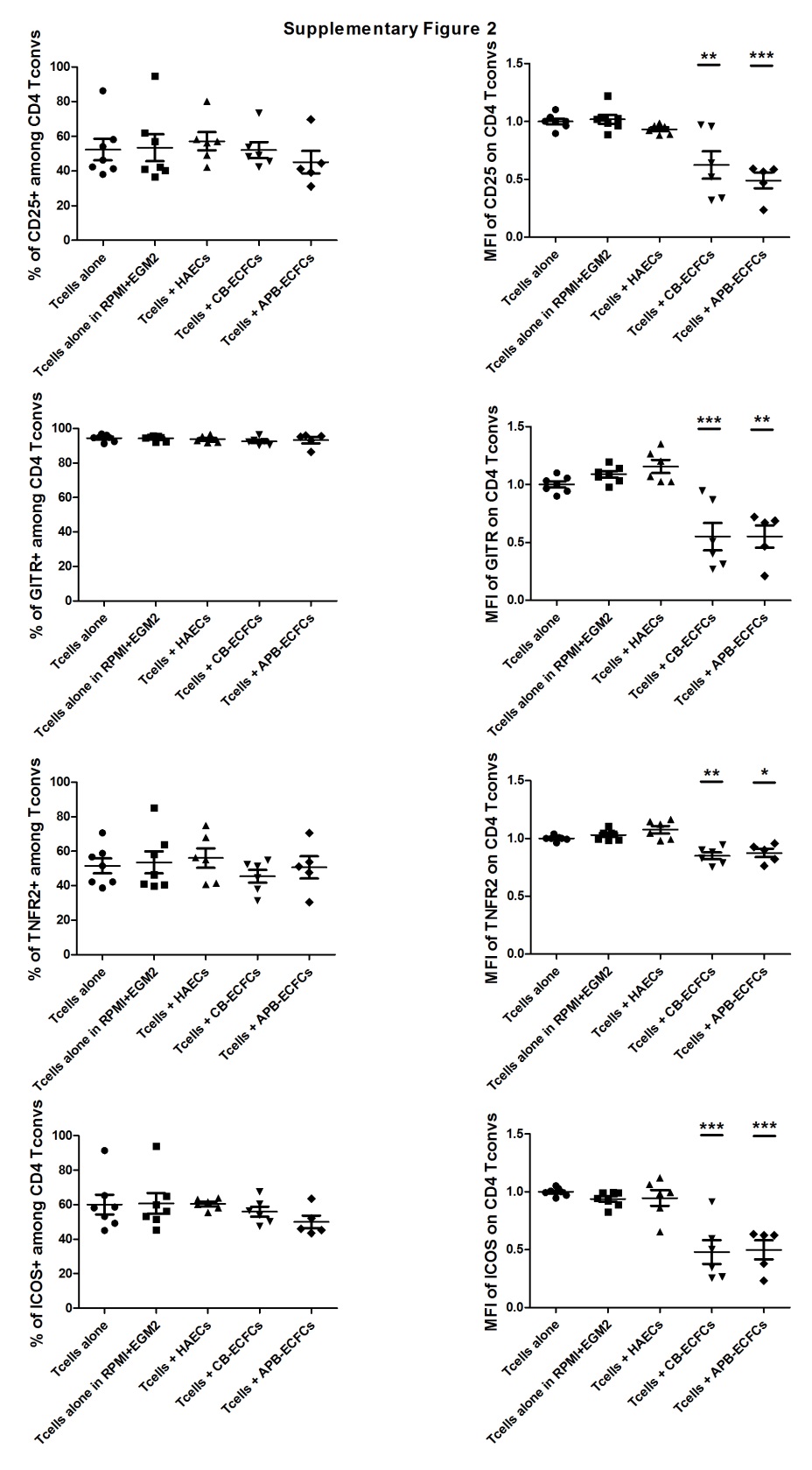
**

**Supplementary Figure 2:**

**ECFCs can modulate CD4^+^ T cell activation markers.** Anti-CD3/CD28 activated CD3^+^CD25^-^ effector T cells were co-cultured with HAECs, CB-ECFCs and APB-ECFCs in fixed 1/6 ratio. After 1 day, T cells were collected and different activation markers were studied. Cells were gated on CD4^+^Foxp3^-^ conventional T cells. For each marker the strategy of gating is indicated on the left and down of the figure. Each dot represents a measured value (n=7 for Tcells alone, Tcells alone in RPMI+EGM2 media and n=6 for Tcells + HAECs, Tcells + CB-ECFCs and n=5 for Tcells + APB-ECFCs). For each group of values, horizontal lines represent mean value and standard error of the mean. MFI values have been normalized with T cells alone control group. One way ANOVA analysis was performed to generate P values. ns: non-significant, *P<.05, **P<.01, ***P<.001.


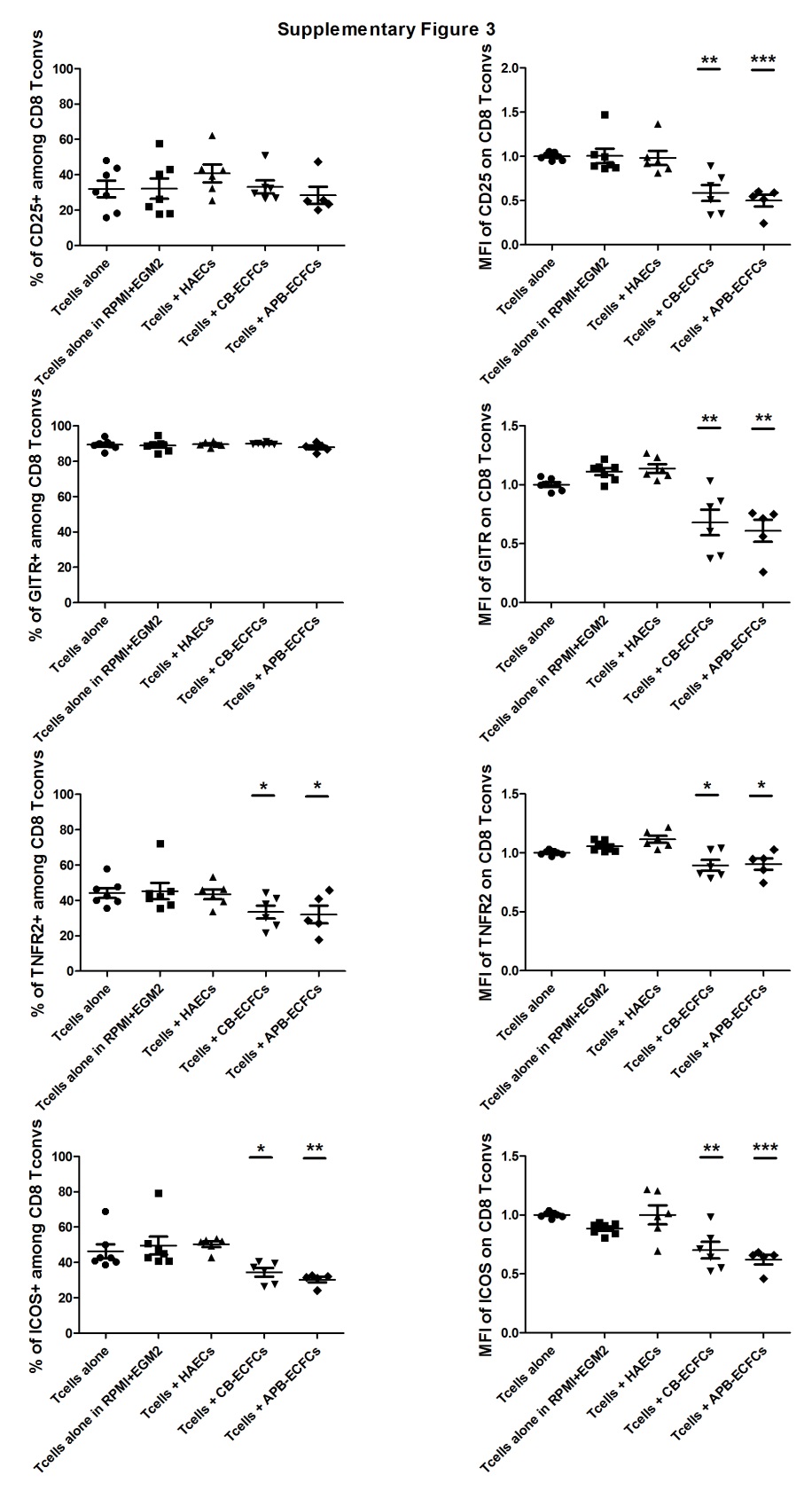


**Supplementary Figure 3:**

**ECFCs can modulate CD8^+^ T cell activation markers.** Anti-CD3/CD28 activated CD3^+^CD25^-^ effector T cells were co-cultured with HAECs, CB-ECFCs and APB-ECFCs in a fixed 1/6 ratio. After 1 day, T cells were collected and different activation markers were studied. Cells were gated on CD8^+^Foxp3^-^ conventional T cells. For each marker the strategy of gating is indicated on the left and down of the figure. Each dot represents a measured value (n=7 for Tcells alone, Tcells alone in RPMI+EGM2 and n=6 for Tcells + HAECs, Tcells + CB-ECFCs and n=5 for Tcells + APB-ECFCs). For each group of values, horizontal lines represent mean value and standard error of the mean. MFI values have been normalized with T cells alone control group. One way ANOVA analysis was performed to generate P values. ns: non-significant, *P<.05, **P<.01, ***P<.001.

**
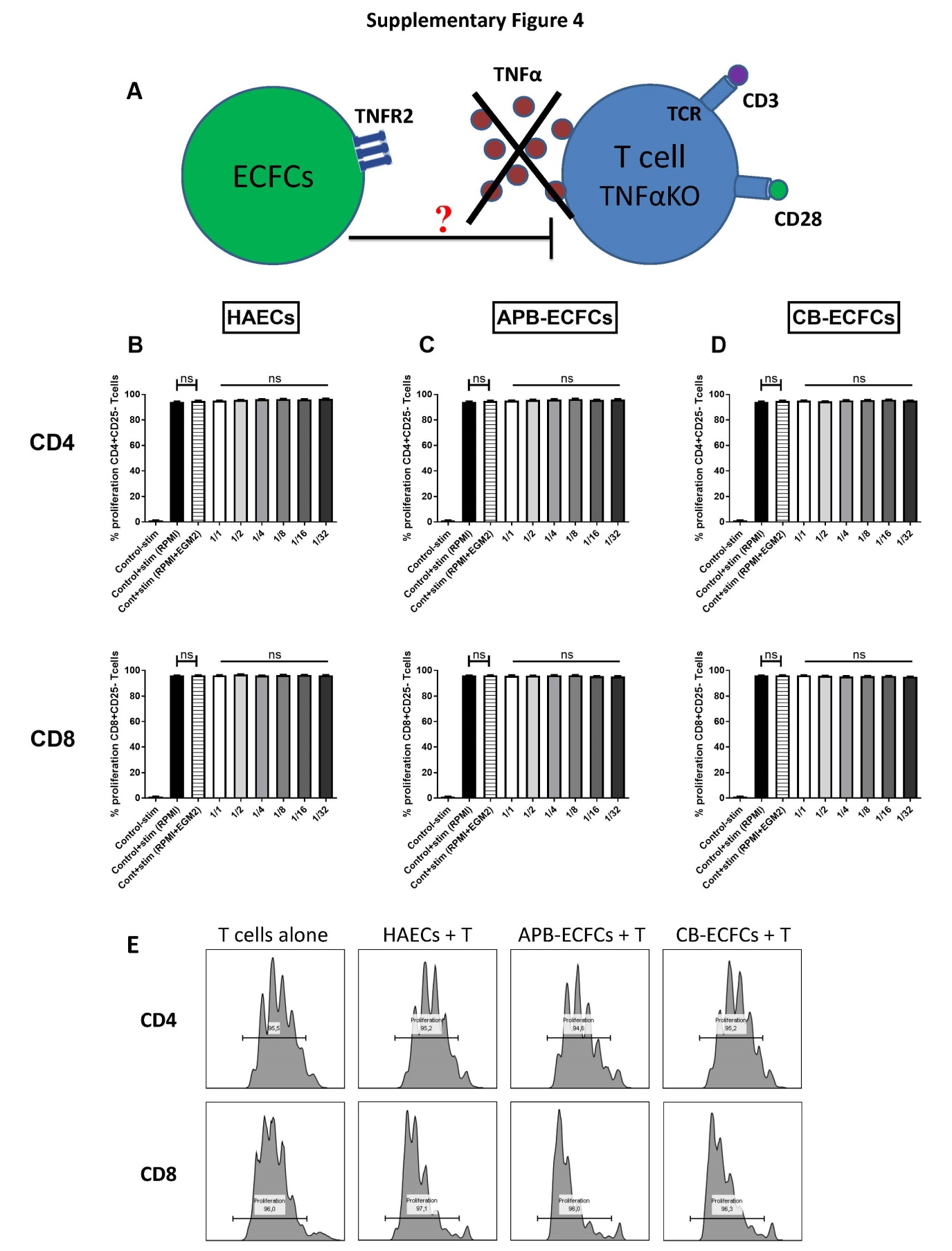
**

**Supplementary Figure 4:**

**ECFCs immunosuppressive effect is entirely diminished when T cells are incapable of TNFα production.** This schematic depicts our hypothesis based on the direct involvement of TNFα/TNFR2 axis in immunomodulatory functions observed by ECFCs. (A) Here, we have interfered with this signaling pathway using T cells harvested from TNFα KO mice that are incapable of TNFα production. In this setting TNFR2 receptor is expressed by ECFCs but no TNFα (ligand) will be produced by T cells. Then, after creating these two manipulated co-culture conditions, we assessed if ECFCs are still able to exert their immunosuppressive function on T cells. Anti-CD3/CD28 activated CFSE^+^CD3^+^CD25^-^ effector TNFα KO T cells were co-cultured with (B) HAECs, (C) APB-ECFCs and (D) CB-ECFCs in 6 different ECs/T cell ratios (n=6). Proliferation of CD4^+^ CD25^-^ T cells (upper graphs) and CD8^+^ CD25^-^ T cells (lower graphs) was measured by flow cytometry. The first bar represents the unstimulated T cells alone (Control-stim, n=4), the second bar represents the anti-CD3/CD28 stimulated T cells alone in RPMI medium (Control+stim (RPMI), n=4), the third bar represents the stimulated T cells alone in 50% RPMI+50% EGM2 media (Cont+stim (RPMI+EGM2), n=4). (E) Flow cytometry representative of proliferation assay. One can see no difference in intensity of CFSE fluorescence among T cell alone, HAECs + T cells , APB-ECFCs + T cells and CB-ECFCs + T cells groups interpreted as no difference in T cell proliferation capacity in absence of TNFα. Data are represented as mean value ± SEM. One way ANOVA analysis was performed to generate P values. ns: non-significant, *P<.05, **P<.01, ***P<.001. Stim: Anti-CD3 and anti-CD28 activation Beads. TCR= T cell receptor.

**

**

**Supplementary Figure 5:**

**Expression of TNFR1 and TNFR2 on different endothelial cells.** Basal expression level of two receptors of TNFα, TNFR1 and TNFR2, was assessed on HAECs and CB-ECFCs by flow cytometry analysis (n=4). One can see higher expression of TNFR2 on CB-ECFCs compared to HAECs and a more elevated expression of TNFR1 on HAECs. For each group of values, horizontal lines represent mean value and standard error of the mean. MFI values have been normalized with HAECs group. Unpaired Student *t* test analysis was performed to generate P values. ns: non-significant, *P<.05, **P<.01, ***P<.001.
