# Supplementary figures and images for: The TNF/TNFR2 signaling pathway is a key regulatory factor in endothelial progenitor cell immunosuppressive effect
Source: Cell Commun Signal. 2020 Jun 16;18:94. doi: 10.1186/s12964-020-00564-3 (PMC7298859; doi:10.1186/s12964-020-00564-3)

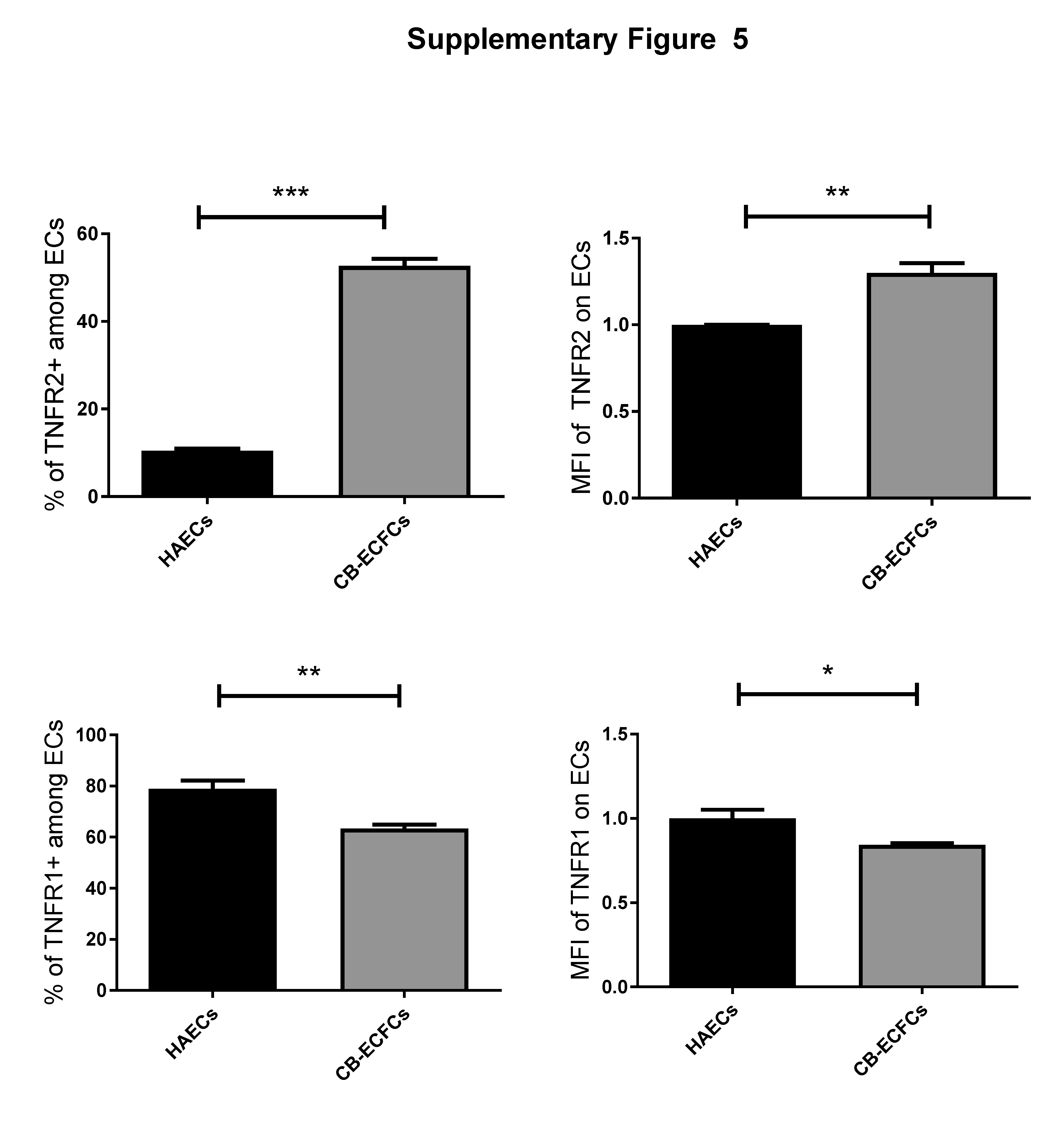

Supplement: Supplementary file 2 — Additional file 1: Supplementary Figure 1. Flow cytometry representative of proliferation assay. Supplementary Figure 2. ECFCs can modulate CD4+ T cell activation markers. Supplementary Figure 3. ECFCs can modulate CD8+ T cell activation markers. Supplementary Figure 4. ECFCs immunosuppressive effect is entirely abolished when T cells are incapable of TNFα production. Supplementary Figure 5. Expression of TNFR1 and TNFR2 on different endothelial cells. [file 12964_2020_564_MOESM1_ESM.zip › Sup 5.tiff]

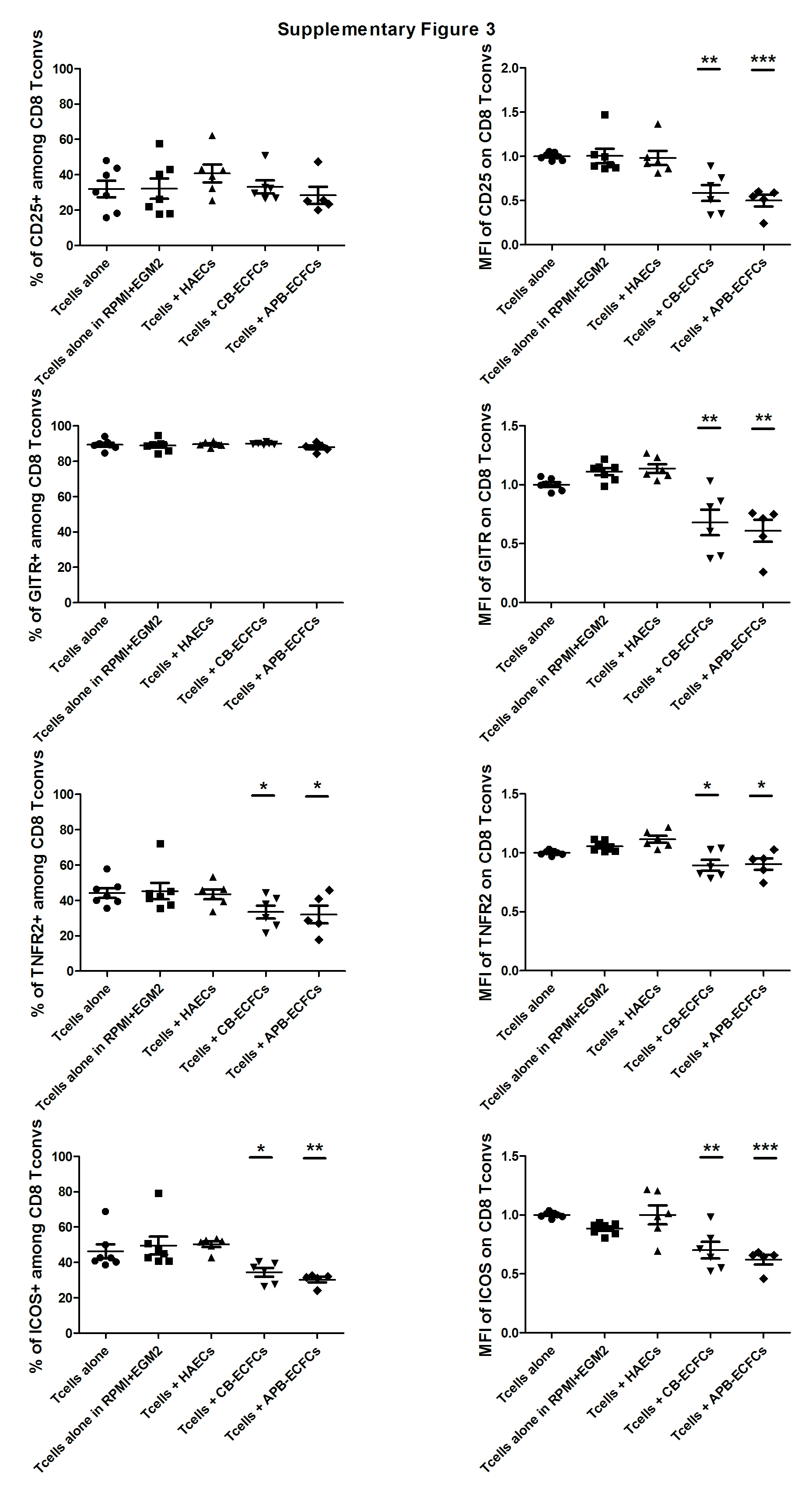

Supplement: Supplementary file 2 — Additional file 1: Supplementary Figure 1. Flow cytometry representative of proliferation assay. Supplementary Figure 2. ECFCs can modulate CD4+ T cell activation markers. Supplementary Figure 3. ECFCs can modulate CD8+ T cell activation markers. Supplementary Figure 4. ECFCs immunosuppressive effect is entirely abolished when T cells are incapable of TNFα production. Supplementary Figure 5. Expression of TNFR1 and TNFR2 on different endothelial cells. [file 12964_2020_564_MOESM1_ESM.zip › Sup 3.tif]

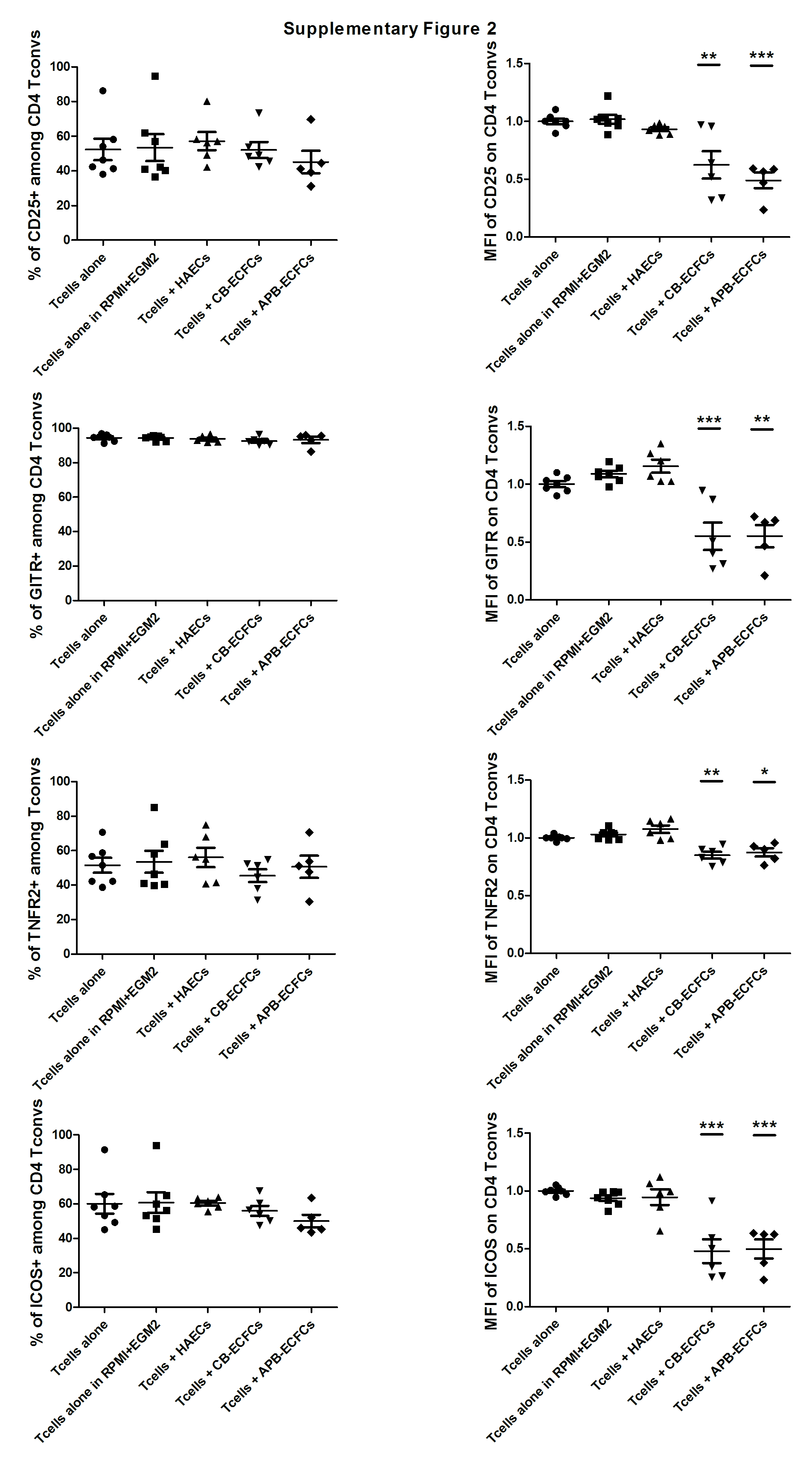

Supplement: Supplementary file 2 — Additional file 1: Supplementary Figure 1. Flow cytometry representative of proliferation assay. Supplementary Figure 2. ECFCs can modulate CD4+ T cell activation markers. Supplementary Figure 3. ECFCs can modulate CD8+ T cell activation markers. Supplementary Figure 4. ECFCs immunosuppressive effect is entirely abolished when T cells are incapable of TNFα production. Supplementary Figure 5. Expression of TNFR1 and TNFR2 on different endothelial cells. [file 12964_2020_564_MOESM1_ESM.zip › Sup 2.tif]

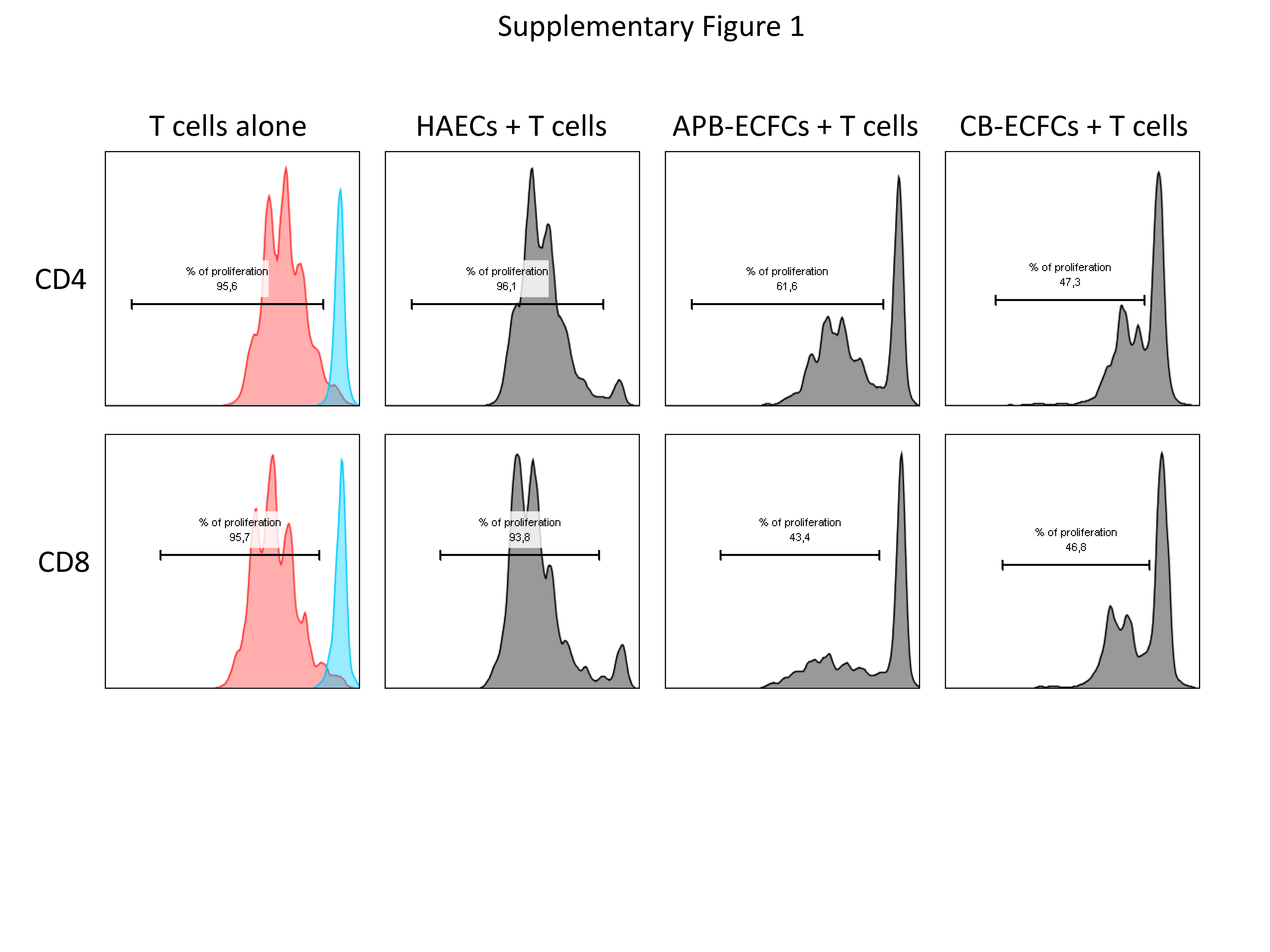

Supplement: Supplementary file 2 — Additional file 1: Supplementary Figure 1. Flow cytometry representative of proliferation assay. Supplementary Figure 2. ECFCs can modulate CD4+ T cell activation markers. Supplementary Figure 3. ECFCs can modulate CD8+ T cell activation markers. Supplementary Figure 4. ECFCs immunosuppressive effect is entirely abolished when T cells are incapable of TNFα production. Supplementary Figure 5. Expression of TNFR1 and TNFR2 on different endothelial cells. [file 12964_2020_564_MOESM1_ESM.zip › Sup 1.tiff]
